# Does the Implementation of Official User Charges Help to Eradicate Informal Payments – Lessons to be Learnt from the Hungarian Experience

**DOI:** 10.3389/fpubh.2015.00181

**Published:** 2015-07-17

**Authors:** Petra Baji, Milena Pavlova, László Gulácsi, Wim Groot

**Affiliations:** ^1^Department of Health Economics, Corvinus University of Budapest, Budapest, Hungary; ^2^Center for Economic Research and Graduate Education – Economics Institute (CERGE-EI), Prague, Czech Republic; ^3^Department of Health Services Research, CAPHRI School for Public Health and Primary Care, Faculty of Health, Medicine and Life Sciences, Maastricht University Medical Center, Maastricht University, Maastricht, Netherlands; ^4^Topinstitute for Evidence-Based Education Research (TIER), Maastricht University, Maastricht, Netherlands

**Keywords:** informal patient payments, informal payments, co-payment, policy analysis, Hungary, out-of-pocket payments, household expenditure

## Introduction – Implementation of Official User Fees to Eradicate Informal Payments

User fees for health care services are a frequently applied policy tool to improve efficiency by controlling demand and contain costs. It is also applied to increase resources for health care, especially when public resources are limited or the increase of income-related contributions is not feasible or not desirable. In addition, in Central and Eastern European (CEE) countries, where informal payments are widespread, the introduction of user fees is often promoted by politicians and policy makers as a potential policy tool to eradicate or “formalize” informal payments (1).

Informal payments are unofficial cash payments or gifts in kinds directly given to the health care personnel before or after using health care services. The literature on informal payments suggests that these payments violate the idea of transparency in health care financing and the accountability of providers, which results in an inefficient use of health care resources and inequalities in access to health care Furthermore, informal payments create adverse incentives for their beneficiaries, which can be in conflict with the government’s policy objectives. (2).

Thus, in the last decades in the CEE countries, several policies have been implemented to eradicate these payments, including the introduction of official user fees for health care services. However, there is no evidence that supports this expectation. On the contrary, there is evidence to suggest that patients continue to pay informally after the introduction of user fees (3–5). In this case, user fees induce a double financial burden on patients, aggravating the existing problems caused by the informal payments. This makes user fees even more unpopular among the public.

According to the OECD Health Statistics in Hungary, the total health expenditure accounts for 8% of the GDP in 2012, with a per capita expenditure of 1,358 USD, which is lower than the OECD average. The share of out-of-pocket payments in total health expenditure has increased considerably during the last decades, and has reached 28.3% in 2012, and accounts for about 5% of the total household expenditure. This level is one of the highest among European OECD countries. Above 80% of these payments are co-payments for pharmaceuticals (80%). There are no official co-payments for health care services, however informal payments are widespread. Hungarian health care consumers have been paying informally for medical services on a routine basis, especially in in-patient care. According to a study carried out in 2010 around one-fifth of the respondents who used physician services during the last 12 months, made informal payments, on average 60 €, and almost half of the respondents who were hospitalized during the last 12 months, paid informally on average 130 € (6). Studies suggest that the scale of these payments has not changed considerably during the last two decades. (7).

In 2007 in Hungary, official user fees were introduced for the use of health care services covered by the social health insurance. The introduction of these fees was one element of the reform package to contain health care cost. According to the documented policy objectives, it was expected that official fees will contribute to the eradication of informal payments (8).

Although the amount of user fees was relatively low (about 1 € per physician visit and per day spent in hospital), the implementation met with strong political opposition and unpopularity among the public. One year later, in April 2008, the payments were abolished as a result of a population referendum initiated by the opposition party in the parliament, where more than 80% of the voters supported the abolishment of the fees (8).

The introduction and abolishment of users fees in Hungary can be considered a natural experiment, and enable us to study the association of formal and informal charges. The aim of this paper is to summarize findings on this issue based on studies carried out to examine the Hungarian case, and draw some policy conclusions.

## Unclear Equity Effects – Decrease of Informal Payments in Low-Income Households

Studying Hungarian households’ expenditure on formal and informal charges during a 4-year period (2005–2008) including the implementation and abolishment of user charges, Baji et al. found, that the co-payments lead to a relatively greater burden on worse-off households. [The Kakwani-index of formal household expenditure for health care services decreased from 0 to −0.1 in 2007 (9)]. At the same time, the study suggests that the increase of formal payments was partly compensated by the decrease of informal payments, especially in low-income households.

During the 1-year period (2007) when user fees were charged for the utilization of health care services, household expenditure on informal payments decreased and became less regressive, i.e., the decrease of informal payments was higher in lower-income households (the share of informal payments decreased from 0.56 to 0.26% of the household income in the poorest quintile, and from 0.20 to 0.14% in the richest quintile). The reduction of informal payments can partly be explained by the drop in the utilization of services, which was recorded during this period. However, a higher decrease in the expenditure on informal payments among low-income households was observed, which also suggests that worse-off households tried to compensate the increase of formal payments by decreasing their expenditure on informal ones.

In another study, Baji et al., analyzed the short-term changes in the probability of paying informally for in-patient and out-patient care 2 months after the implementation of user fees. The findings suggest that the probability of paying informally decreased only for hospitalization among elderly people (10). Elderly with a lower ability to pay respond to the increased burden of formal payments with a decrease of their expenditure on informal payments, as elderly people are likely to be among lower-income households in Hungary.

Overall, the results presented above suggest that the implementation of user fees can lead to a reduction of informal payments of those health care consumers who are not able to meet the double burden. However, this finding raises questions concerning equal access to health care services. It is possible that consumers, who are not able to pay informally for health care services, may not obtain the services that other consumers obtain because they continue to pay. In this way, the reduction of informal payments can create even greater inequalities between different income groups.

## Complements or Substitutes?

Nevertheless, both studies show that informal payments remain relevant. For those who are able to pay and have no budget-constraints, formal payments do not substitute for informal payments, at least at the short term, these payments rather co-exist and complement each other.

Findings from a qualitative study provide some explanation for this (11). Consumers argue that neither the measure nor the objectives, nor the beneficiary of informal payments is the same as those of user fees. Formal and informal payments serve different objectives, and consumers achieve different benefits by paying formally and informally. In Hungary, informal payments are directly pocketed by physicians (mostly by the head/manager) and contribute to their income. In this way, these payments may affect the choice of the physician, the attitude of the personnel, or in some cases, even the access to services. On the other hand, formal payments have more potential when used to improve equipment or the maintenance of health care facilities or if otherwise reinvested in health care provision. However, according to public opinion, the user fees introduced in 2007 did not contribute to the improvement of service quality (11).

Also, attributes connected to the health care personnel (i.e., the skills and reputation of the physician, as well as the attitude of the health care personnel) are more important for health care consumers than other service attributes, like the health care facility, equipment or even waiting time for the visits, or traveling time to the health care facility (12). These results indicate the importance of the patient–physician relationship. Since informal payments are a relevant element of this relationship, it is quite probable that despite the formal charges, patients continue to pay informally directly to their physician, as an expression of their gratitude or in the hope of getting extra services, better access or more personal attention. Or, they keep on paying informally, because of the fear that they do not obtain the care they need if they do not pay.

Furthermore, Hungarian health care consumers are rather tolerant toward informal payments, mainly because these payments provide additional salary to the (underpaid) health care personnel (2). Also, some population groups (e.g., elderly people and consumers from the capital) seem to prefer paying informally for health care services (6).

To summarize, health care consumers doubt that user fees in 2007 had the potential to eradicate informal payments as formal fees do not substitute for informal payments. The lack of support for the stated policy objectives makes the implementation of these charges more unpopular and this might be one of the reasons why the fees were rejected by the population in the referendum. Findings suggest that the problem of informal payments remains relevant even after the implementation of user fees. However, if formal payments do not substitute for informal payments, user fees induce a double financial burden on health care consumers, and make official charges less acceptable to the public.

## Policy Implications of the Findings

After the implementation of user fees, informal payments remain relevant and the two payments co-exist. However, the increase of formal payments may indeed lead to a decrease in informal payments, specifically among low-income households who are not able to pay for the double (formal and informal) price.

Regarding the policy implications, we can say that the reduction of informal payments is a desirable policy aim because of the adverse effect of these payments on the health care system, i.e., inefficient use of resources, barrier to access, and adverse financial incentives for beneficiaries. However, if the decrease of these payments only occurs among the lower-income households, among those who are not able to pay for the double burden, this mechanism can increase inequalities in access among households with different income. Those who are able to pay for the double burden may have better access to health care services compared to those who are not able to pay. Thus, the implementation of user fees is hardly sufficient and rather a controversial policy tool to diminish informal payments.

The Hungarian experience with the implementation of user fees confirms that public acceptance is crucial for a successful implementation of user fees. However, in Hungary the declared policy objectives of the implementation of the user fees were not supported by the public, making the implementation of these charges more unpopular.

## Conflict of Interest Statement

The authors declare that the research was conducted in the absence of any commercial or financial relationships that could be construed as a potential conflict of interest.
